# Marker-aided Incorporation of *Xa38*, a Novel Bacterial Blight Resistance Gene, in PB1121 and Comparison of its Resistance Spectrum with *xa13* + *Xa21*

**DOI:** 10.1038/srep29188

**Published:** 2016-07-11

**Authors:** Ranjith K. Ellur, Apurva Khanna, Gopala Krishnan. S, Prolay K. Bhowmick, K. K. Vinod, M. Nagarajan, Kalyan K. Mondal, Nagendra K. Singh, Kuldeep Singh, Kumble Vinod Prabhu, Ashok K. Singh

**Affiliations:** 1 Division of Genetics, ICAR-Indian Agricultural Research Institute, New Delhi, India; 2 RBGRC, ICAR-Indian Agricultural Research Institute, Aduthurai, Tamil Nadu, India; 3 Division of Plant Pathology, ICAR-Indian Agricultural Research Institute, New Delhi, India; 4 ICAR-National Research Centre on Plant Biotechnology, New Delhi, India; 5 School of Agricultural Biotechnology, Punjab Agricultural University, Ludhiana, India

## Abstract

Basmati rice is preferred internationally because of its appealing taste, mouth feel and aroma. Pusa Basmati 1121 (PB1121) is a widely grown variety known for its excellent grain and cooking quality in the international and domestic market. It contributes approximately USD 3 billion to India’s forex earning annually by being the most traded variety. However, PB1121 is highly susceptible to bacterial blight (BB) disease. A novel BB resistance gene *Xa38* was incorporated in PB1121 from donor parent PR114-*Xa38* using a modified marker-assisted backcross breeding (MABB) scheme. Phenotypic selection prior to background selection was instrumental in identifying the novel recombinants with maximum recovery of recurrent parent phenome. The strategy was effective in delimiting the linkage drag to <0.5 mb upstream and <1.9 mb downstream of *Xa38* with recurrent parent genome recovery upto 96.9% in the developed NILs. The NILs of PB1121 carrying *Xa38* were compared with PB1121 NILs carrying *xa13 + Xa21* (developed earlier in our lab) for their resistance to BB. Both NILs showed resistance against the Xoo races 1, 2, 3 and 6. Additionally, *Xa38* also resisted Xoo race 5 to which *xa13* + *Xa21* was susceptible. The PB1121 NILs carrying *Xa38* gene will provide effective control of BB in the Basmati growing region.

Harmonious combination of cooking quality characteristics and pleasant aroma is the uniqueness of Basmati rice. Intensive program on genetic improvement of Basmati rice has led to the development of a landmark rice variety, Pusa Basmati 1121 (PB1121), at ICAR-Indian Agricultural Research Institute (IARI), New Delhi. This is a medium duration high-yielding Basmati rice variety characterized by extra-long slender grains with very high kernel length after cooking (22–25 mm), high cooked kernel elongation ratio (2.5), intermediate amylose content, high volume expansion after cooking (>4 times) and strong aroma^1^. These unique properties of PB1121 have attracted consumers worldwide. PB1121 currently occupies 1.35 million hectares, which is nearly 70% of the area under Basmati cultivation in India and contributes approximately 4 million tons to Basmati rice production annually. With the recognition of PB1121 as Basmati during 2008, the forex earning of the country has risen from USD 0.67 million to USD 4.5 billion in 2014–2015 2, in which the contribution of PB1121 is about 65%. This variety has not only revolutionized the international Basmati rice trade but also improved the livelihood of millions of Basmati farmers. However, major weakness of PB1121 is its susceptibility to a number of diseases such as to bacterial blight (BB)^3^, blast^3^ and bakanae. The BB disease is caused by a gram-negative bacteria, *Xanthomonas oryzae* pv. *oryzae* (Xoo), which severely constrains rice productivity and quality. Xoo breaches the plant epidermis and multiplies in the xylem vessels which lead to disease symptoms. The pathogen produces various virulence factors including EPS, extracellular enzyme, iron chelating siderophores and the type III-secretion dependent effectors, which are collectively essential for virulence^4^,^5^,^6^,^7^,^8^ and to counterattack plant’s immune response termed as PAMP-triggered immunity^9^. Xoo populations are highly diversed in terms of mode of action through virulence factors^10^,^11^,^12^,^13^,^14^,^15^. Mondal *et al*.^15^ characterized 780 Xoo isolates collected from thirteen states of India and grouped them into six races based on their reaction to 12 near isogenic lines (NILs). To date, 30 races of Xoo have been documented globally^16^,^17^,^18^.

Utilizing the host plant resistance is an effective means to manage this pathogen in rice. As of now, 41 BB resistance genes have been reported among which 8 have been cloned and characterized^3^,^19^,^20^. However, four genes namely, *Xa4*, *Xa8*, *xa13* and *Xa21* have been reported to be effective against the Xoo isolates prevalent in Basmati growing regions of India^21^. Furthermore, the combination of genes *xa13* + *Xa21*, *xa5* + *xa13* + *Xa21* and *Xa4* + *xa5* + *xa13* + *Xa21* were reported to be equally effective and has provided greater degree of resistance as compared to monogenic lines. Therefore, *xa13* + *Xa21* have been widely used in the Basmati rice improvement program which resulted in development of several BB resistant genotypes such as Improved Pusa Basmati 1 22,23, Pusa 1718 and Pusa 1728 3. Extensive utilization of these genes over a long period of time, may lead to resurgence of pathogen with virulence genes that can overcome this combination. Therefore, deployment of diverse genes is essential to prolong the durability of resistance genes and to counter the development of virulent races of the pathogen.

Recently, a novel BB resistance gene *Xa38* was identified in *Oryza nivara* and mapped on to the long arm of chromosome 4 24. It was further fine mapped to a region of 38.4 kb and an InDel marker based on the putative candidate gene LOC_Os04gg53050-1 was developed, which co-segregated with the BB resistance^25^. It is expected that deployment of *Xa38* in rice varieties may help in checking the breakdown of resistance to Xoo. Therefore, the present study was undertaken to transfer *Xa38* into the elite Basmati rice variety PB1121 through marker assisted backcross breeding (MABB) and to study its resistance spectrum in comparison to the combination of genes *xa13* + *Xa21* in the genetic background of PB1121.

## Materials and Methods

### Marker assisted breeding scheme

**Figure 1:**
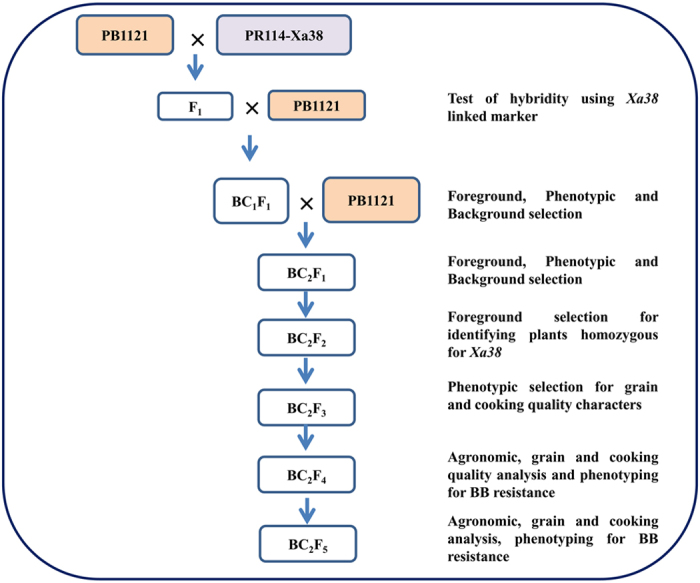
Marker assisted backcross breeding scheme adopted for introgression of *Xa38* in Pusa Basmati 1121.

PR114-*Xa38*, an introgression line generated from a cross PR114/*Oryza nivara* carrying the BB resistance gene *Xa38* 24 was used as the donor parent and PB1121 was used as the recurrent parent in a MABB (Fig. 1). Individual F_1_ plant generated from the cross PB1121/PR114-*Xa38* (designated as Pusa 1927), confirmed for hybridity using the *Xa38* linked marker-Os04g53050-1 was backcrossed with the recurrent parent PB1121 to generate BC_1_F_1_ seeds. Foreground selection followed by phenotypic and background selection was carried out to identify the plants with maximum recovery for recurrent parent phenome (RPP) and genome (RPG). The best BC_1_F_1_ plant was further backcrossed to generate BC_2_F_1_ seeds. The BC_2_F_1_ plants were also subjected to foreground, phenotypic and background selection to identify the plant with maximum recovery for RPG and RPP. The superior plant was advanced to BC_2_F_2_ generation and foreground selection was repeated in order to identify the plants homozygous for *Xa38*. Further, these plants were advanced via pedigree based phenotypic selection to obtain *Xa38* introgressed near isogenic lines (NILs) of PB1121.

### Molecular Marker Analysis

DNA from each of the leaf sample was extracted using the modified protocol of Murray and Thompson^26^. PCR reaction was carried out using 20 ng of template DNA, 1x PCR buffer [10 mM Tris–HCl (pH 8.4); 50 mMKCl], 5 pmol of each primer, 0.05 mM dNTPs, 1.8 mM MgCl_2_ and 1U of Taq DNA polymerase (Invitrogen, Life Technologies, Brazil). The PCR was carried out in a G-Storm thermal cycler with the following program: 1) initial denaturation at 94 °C for 5 min; 2) 35 cycles for denaturation for 30 s at 94 °C, annealing for 30 s at 56 °C, extension for 1 min at 72 °C; and 3) final extension at 72 °C for 7 min. The amplified product was resolved in a gel electrophoresis using 3.5% Metaphor^TM^ Agarose gel and visualized on GelDoc^TM^ XR (Bio-Rad Laboratories Inc., USA).

Foreground selection was carried out using the *Xa38* linked molecular marker-Os04g53050-1 25. For background selection, 628 genome-wide SSR markers were used in parental polymorphism survey between the parental lines PB1121 and PR114-*Xa38*, and a total of 70 polymorphic markers spanning uniformly across the genome were selected. Recombinant selection was carried out using the markers RM5511 and RM17523 flanking the BB resistance gene *Xa38* on chromosome 4.

### Screening for resistance to BB

The NILs, along with the checks PB1121 and PR114-*Xa38*, were grown in field conditions till maximum tillering stage. Different races of Xoo maintained as single spore culture was used to generate the BB suspension with a density of 10^9^ cells/mL. Top five leaves from each of the entry were inoculated with Xoo isolate through the clip inoculation method^27^. After 21 days of inoculation, the BB lesion length was measured using the disease assessment scheme as adopted by Mondal *et al*.^14^, wherein, lesion length of <5 cm was considered resistant, 5–10 cm was considered moderately resistant, 10–15 cm was considered moderately susceptible and more than 15 cm was considered highly susceptible reaction. Additionally, the resistance spectrum of Pusa1927 (PB1121 + *Xa38*) was compared with Pusa1718 (PB1121 + *xa13* + *Xa21*) using a set of six races of Xoo.

### Agronomic and Cooking Quality Evaluation

The NILs, along with the recurrent and donor parents, were evaluated for agronomic traits in randomized complete block design (RCBD) with three replications. Standard agronomic management practices were followed for raising the rice crop at ICAR-IARI, New Delhi. The data was recorded on five plants from each of the entries for the characters namely: days to 50% flowering (DFF), plant height (PH), panicle length (PL), filled grains per panicle (FGP), spikelet fertility (SF), thousand grain weight (TGW) and grain yield (GY). Further, the lines were also analyzed for grain and cooking quality parameters such as kernel length before cooking (KLBC), kernel breadth before cooking (KBBC), length/breadth ratio (L/B), kernel length after cooking (KLAC), kernel breadth after cooking (KBAC) and aroma as described by Khanna *et al*.^28^.

## Results

### Development of PB1121-NILs carrying BB resistance gene *Xa38*

The F_1_s generated from the cross PB1121/PR114-*Xa38* were tested for hybridity using the marker-Os04g53050-1. A single plant confirmed for hybridity was backcrossed to recurrent parent and a total of 468 BC_1_F_1_ seeds were produced, out of which 223 plants were found to be heterozygous for *Xa38*. All the heterozygous plants were subjected to phenotypic evaluation for agro-morphological traits. Ten superior plants, possessing relatively superior recovery for RPP were selected. The phenotypic selection for key Basmati traits such as KLAC and KBAC resulted in positive selection differential of 0.32 mm and 1.56 mm, respectively. Further, the background selection resulted in RPG recovery ranging from 70.6% to 79.29%. The best BC_1_F_1_ plant with RPG recovery of 79.29% was selected for further backcrossing with the recurrent parent PB1121 and a total of 198 BC_2_F_1_ seeds were produced. Foreground selection revealed that a total of 100 plants were heterozygous for *Xa38*, which were subjected to phenotypic selection for agronomic, grain and cooking quality traits. The selection differential was positive with a gain of 0.12 mm for KLAC and 0.36 mm for KBAC in the selected top ten superior BC_2_F_1_ plants. These plants when subjected to background selection using 29 markers which were heterozygous in the BC_1_F_1_ generation; the RPG recovery ranged from 83.6% to 88.6%.

**Figure 2:**
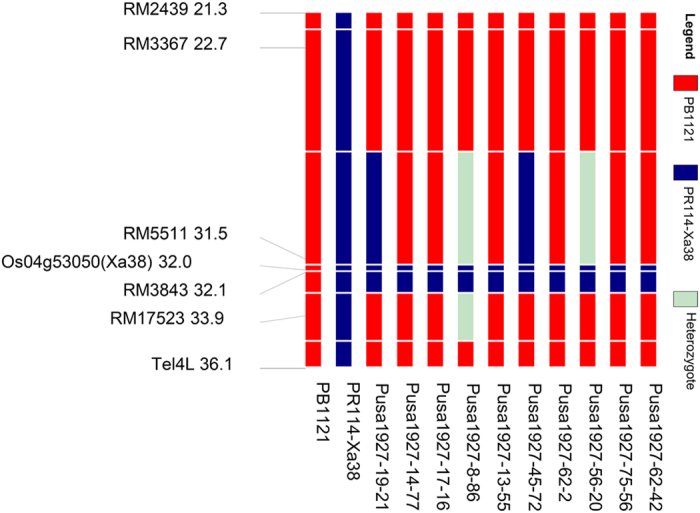
Depiction of extent of linkage drag in the selected NILs carrying BB resistance gene *Xa38*.

The superior BC_2_F_1_ plant was selfed to produce 243 BC_2_F_2_ plants where 65 plants were homozygous for *Xa38*. Based on grain and cooking quality parameters, the selection differential was 0.29 mm in KLAC and 0.15 mm in KLBC. Furthermore, background selection with 16 markers remained heterozygous in BC_2_F_1_ generation, and the RPG recovery increased to the range of 88.57% to 92.86%. Finally, best plant was selected and advanced to BC_2_F_3_ generation. In ensuing BC_2_F_3_ generation, after phenotypic selection for the key Basmati traits KLAC and KLBC, the selection differential was 0.37 mm and 0.02 mm, respectively. The corresponding RPG recovery in this generation ranged from 93.18 to 94.70%. Repeating the same process in the next generation, BC_2_F_4_, the selection differential observed due to selection was 0.45 mm for KLAC and no gain observed for KBAC. The aroma score did not show significant variation between generations from that of PB1121 except in backcross generations (BC_1_F_1_ and BC_2_F_1_). The linkage drag was delimited to the region of <0.5 mb upstream and <1.9 mb downstream the BB resistance gene *Xa38* (Fig. 2). Finally, ten best BC_2_F_5_ families were isolated and evaluated for agronomic and cooking quality characters. The number of plants generated during each of the backcross generations, the selection differential obtained due to phenotypic selection and the background recovery realized are presented in Table 1.

### Evaluation of NILs for Agronomic and Cooking Quality Performance

**Figure 3:**
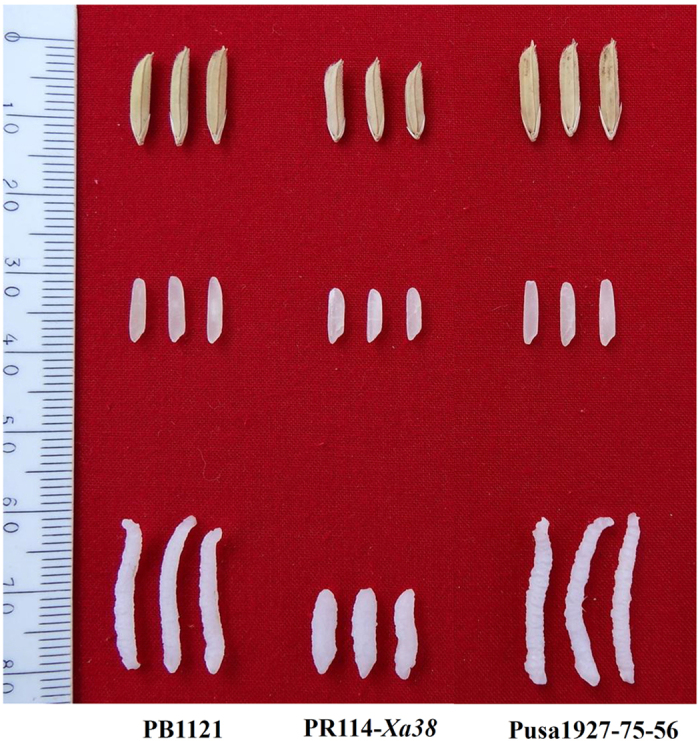
Grain and cooking quality of the recurrent parent PB1121, donor parent PR114-Xa38 and the PB1121-NIL (Pusa1927-75-56) possessing *Xa38*.

All the NILs tested were found to be at par with the recurrent parent PB1121 with some exceptions such as NIL Pusa1927-17-16 which was found to mature 2 days later with slightly taller stature but possessed higher spikelet fertility than that of PB1121 (Table 2). Interestingly, six NILs, namely Pusa1927-19-21, Pusa1927-14-77, Pusa1927-8-86, Pusa1927-13-55, Pusa1927-45-72 and Pusa1927-62-2, were found to possess significantly higher spikelet fertility as compared to PB1121. The NIL Pusa1927-8-86 was found to be significantly superior in yield as compared to PB1121 owing to its better spikelet fertility. With respect to grain and cooking quality, all NILs were at par with the recurrent parent PB1121 (Table 3). However, three NILs Pusa1927-19-21, Pusa1927-14-77 and Pusa1927-17-16 were found to be significantly inferior, while NIL Pusa1927-75-56 was significantly superior as compared to the recurrent parent PB 1121 for the trait kernel length after cooking (Table 3 and Fig. 3).

### Comparative Analysis of Resistance Spectrum of BB Resistance Genes, *xa13* + *Xa21* and *Xa38*

**Figure 4:**
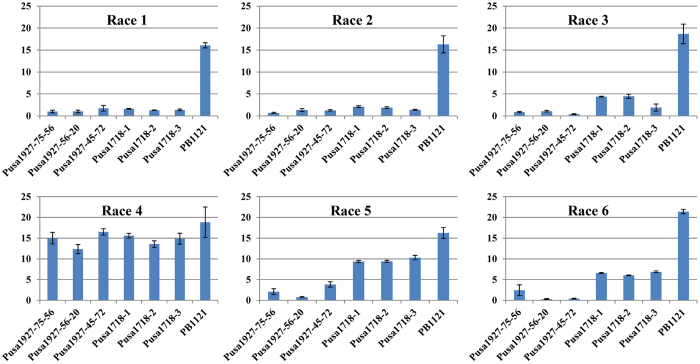
Bacterial blight disease score of NILs carrying *xa13 + Xa21* and *Xa38* in the genetic background of Pusa Basmati 1121. Legend: PB1121 - Pusa Basmati 1121, Pusa 1927-PB1121 + *Xa38* and Pusa 1718-PB1121 + *xa13 + Xa21*.

The resistance imparted by the BB resistance genes, *xa13* + *Xa21* and *Xa38* was compared by subjecting the NILs Pusa1927 (PB1121 + *Xa38*) and Pusa1718 (PB1121 + *xa13* + *Xa21*) against six different Xoo isolates. The disease reaction observed in the recurrent parent PB1121, Pusa1927 (PB1121 + *Xa38*) and Pusa1718 (PB1121 + *xa13 + Xa21*) is presented in Table 4 and Fig. 4.

The recurrent parent PB1121 was found to be highly susceptible to all the six Xoo races, while Pusa1927 (PB1121 + *Xa38*) and Pusa 1718 (PB1121 + *xa13* + *Xa21*) were found to be highly resistant against Xoo races 1, 2, 3 and 6. However, for race 5, Pusa1718 (PB1121 + *xa13 + Xa21*) was found to be moderately resistant to moderately susceptible with the lesion length ranging from 9.37 ± 0.26 cm to 10.30 ± 0.55 cm, while NIL Pusa1927 (PB1121 + *Xa38*) was highly resistant with the lesion length ranging from 0.77 ± 0.15 cm to 3.83 ± 0.64 cm. However, both Pusa1718 and Pusa1927 were found to be susceptible against race 4 of Xoo.

## Discussion

Basmati is the specialty rice of India, acclaimed worldwide for its unprecedented cooking quality characters and appealing aroma. Being susceptible to most of the diseases and pests including BB disease, the most popular Basmati cultivar in India, PB1121, which enjoys an export contribution of about 60–70%, often entreats agro-management practices that include use of chemical pesticides. Clean production of Basmati rice under eco-friendly environments is a prime target for producing export standard rice. Arming the cultivars with in-built resistance is therefore inevitable in contemporary Basmati breeding. Several genes conforming resistance to diseases such as BB and blast have already been deployed in Basmati cultivars^3^,^22^,^28^.

In the current study, PB1121 was incorporated with the novel BB resistance gene *Xa38* using the donor parent PR114-*Xa38*, an introgression line developed by crossing PR114 an elite rice cultivar of Punjab, India with a wild rice (*O. nivara*) acc. IRGC81825 25. PR114 is a semi-dwarf, stiff strawed variety narrow, dark green erect leaves and possess extra-long, clear translucent non-aromatic grains^29^. However, the grain and cooking quality of PR114 and PR114-*Xa38* is quite inferior as compared to the recurrent parent PB1121. Using poor grain quality donor parent in Basmati rice breeding impairs the grain and cooking quality of derived line, which imposes a challenge of recovering the quality of recurrent parent. Therefore, maximization of RPG and RPP recovery in the derived NILs is important^28^.

The recovery of recurrent parent genome to the extent of 96.9% was achieved with two backcrosses through integration of foreground, phenotypic and background selection. Background selection using the SSR markers usually target the non-coding and heterochromatic regions and therefore may not quantify the recovery of functional part of the genome. However, the phenotypic selection, which indirectly targets functionally expressed region of the genome, was augmented for hastening the process of reconstruction of recurrent parent phenotype. It was interesting to note that the selection differential for the trait KLAC was progressive along the generations and the recombinants at par or superior to recurrent parent PB1121, could be obtained by BC_2_F_4_ generation. For the trait KLBC, the selection differential was positive till BC_2_F_3_ generation only; whereas, in case of aroma, the trait fixation was achieved in BC_2_F_1_ generation. The varying selection differential for different traits can be attributed to the number of genes governing them. The trait KLAC and KLBC are under polygenic control governed by additive, dominance and epistatic gene action. Hence, progressive response to selection in later generation was obtained. Similar phenotypic gain was obtained for other agronomic traits also (data not presented). Aroma is a key Basmati trait, which is mainly governed by two recessive genes *badh1*and *badh2* 30,31. Therefore, fixation for this trait was possible in the early backcross generations itself. The NILs carrying *Xa38* gene, were agronomically similar to their recurrent parent PB1121 in most of the cases (Tables 2 and 3). Phenotypic selection prior to background selection has led to isolating the novel recombinant Pusa1927-75-56, which was similar to the recurrent parent but possessed significantly superior KLAC. The success of integrating phenotypic selection along with background selection in the MABB program for isolating superior recombinants and substantially reducing the number of plants for background selection has been demonstrated earlier by Ellur *et al*.^3^. In cases where limited backcrosses are given, NILs derived from a common backcross program differ with respect to their agronomic performance, grain and cooking quality traits because of the presence of minor donor segments on different chromosomes, which offer great opportunity to select NILs excelling the recurrent parent^3^,^32^. It is pertinent to mention that stringent selection for grain quality has resulted in near complete recovery of PB1121 grain quality traits in most of the NILs developed.

The efficacy of phenotypic selection in hastening the recovery of recurrent parent phenotype has been earlier demonstrated while developing Improved Pusa Basmati 1 carrying BB resistance genes *xa13 + Xa21*^21^,^22^ and Pusa 1609 carrying blast resistance genes^32^,^33^. In the current study, the recombinant selection on carrier chromosome resulted in delimiting the linkage drag to <0.5 mb upstream and <1.9 mb downstream of the *Xa38* gene, which otherwise could have been a limiting step because of the donor parent PR114-Xa38, being a non –Basmati variety.

On artificial screening, when all the derived NILs of PB1121 were exposed to two virulent BB isolates along with their parents, the donor PR114-*Xa38* and the PB1121 NILs produced incompatibility reaction, as against PB1121 that was highly susceptible. The BB isolates, IARI-Kaul and IARI-Ludhiana were sourced from Haryana and Punjab respectively, two major Basmati rice growing states of India. Both isolates were the most predominant pathogenic variants of BB in respective states^3^. The resistance imparted by *Xa38* gene implied that the gene may be effective against pathogenic races available in the major Basmati growing regions of India. Effectiveness of *Xa38* against all common pathogenic variants of Xoo in Punjab has been earlier reported by Vikal *et al*.^34^.

In a recent study, Mondal *et al*.^14^ grouped the Xoo isolates of India into six different races based on their reaction against 12 monogenic-NILs carrying different BB resistance genes. However, these monogenic lines did not include *Xa38* gene. The combination of BB resistance genes, *xa13* + *Xa21* has been considered as one of the best combinations that can combat most of the virulent races of the Xoo. To establish the spectrum of BB resistance of *Xa38* gene and to assess its efficacy in relation to *xa13* + *Xa21* combination, a set of *Xa38* introgressed NILs of PB1121 were inoculated with the six Xoo races along with the recurrent parent PB1121. The BB resistance gene *Xa38*, as well as *xa13* + *Xa21*, were effective against the Xoo races 1, 2, 3 and 6. Races 1 and 2 are predominantly distributed in the eastern India; race 3 is prevalent in Haryana; while race 6 is predominant in entire northwestern region. Race 5 reported to be sporadically distributed across India could not infect the NILs with *Xa38* while it was virulent against the PB1121 NILs carrying *xa13* + *Xa21*. Therefore, deployment of *Xa38* into the genetic background of elite rice varieties is essential to effectively combat the Xoo races 1, 2, 3, 5 and 6. On the other hand, Xoo race 4, reported to be present in eastern and north-western states of India, was the most virulent race that could knock down *xa13* + *Xa21* combination as well as *Xa38*. Thus, further efforts are required to identify novel BB resistance genes or gene combinations that can provide resistance against the race 4 of Xoo.

To conclude, the study reports successful introgression of a highly effective resistance gene *Xa38* into the genetic background of an elite Basmati rice variety PB1121 using a modified-MABB. The improved lines are now under evaluation in the National Basmati Trials for subsequent release. The study also highlights the importance of comparing the spectrum of resistance genes either singly or in combination against a wide range of pathogenic races, so that effective deployment of resistance genes can be planned to avoid future outbreak of disease.

## Additional Information

**How to cite this article**: Ellur, R. K. *et al*. Marker-aided Incorporation of *Xa38*, a Novel Bacterial Blight Resistance Gene, in PB1121 and Comparison of its Resistance Spectrum with *xa13* + *Xa21*. *Sci. Rep*. **6**, 29188; doi: 10.1038/srep29188 (2016).

## Figures and Tables

**Table 1:** Number of plants produced, selection differential and recurrent parent genome recovery in backcross generations.

Generation	No. of plants	Foreground Selection	Traits	PB1121	Phenotypic Selection for quality traits				Background Selection
		No. of plants selected			Range before selection	Range in selected top ten plants	Δd	Progressive Δd	RPG recovery (%)
BC_1_F_1_	468	223	KLAC (mm)	18.56	11.26 to 15.65	14.89 to 15.65	0.32	0.32	70.60–79.29
			KLBC (mm)	8.65	6.78 to 7.98	7.11 to 7.98	1.56	1.56	
			Aroma	2	0 to 2	1 to 2	–		
BC_2_F_1_	198	100	KLAC (mm)	18.41	15.00 to 17.20	16.82 to 17.24	0.12	0.44	83.60–88.57
			KLBC (mm)	8.65	7.12 to 8.72	8.21 to 8.72	0.36	1.92	
			Aroma	2	1 to 2	2	–		
BC_2_F_2_	243	65	KLAC (mm)	18.45	17.18 to 18.25	17.95 to 18.25	0.29	0.73	88.57–92.86
			KLBC (mm)	8.75	7.83 to 8.79	8.25 to 8.79	0.15	2.07	
			Aroma	2	2	2	–		
BC_2_F_3_	145	10	KLAC (mm)	18.54	17.25 to 18.82	17.95 to 18.82	0.37	1.10	93.18–94.70
			KLBC (mm)	8.68	8.56 to 8.78	8.64 to 8.76	0.02	2.09	
			Aroma	2	2	2	–		
BC_2_F_4_	25	10	KLAC (mm)	18.66	17.65 to 19.16	18.22 to 19.16	0.45	1.55	93.18–96.92
			KLBC (mm)	8.74	8.26–8.74	8.42 to 8.74	0.00	2.09	
			Aroma	2	2	2	–		

**Δd –** Selection differential, the mean deviation of the selected population from the base population.

**Table 2:** Agronomic performance of the BC_2_F_5_ NILs carrying *Xa38* in the genetic background of PB1121.

Genotype	DFF (days)	PH (cm)	TN	PL (cm)	FGP	SF (%)	TGW (g)	YLD (kg/ha)
Pusa1927-19-21	104.5	105.34	14.47	28.11	106.73	90.48*	28.71	5454
Pusa1927-14-77	105.0	104.04	15.38	29.53	110.01	90.91*	27.42	6292
Pusa1927-17-16	107.0*	109.77	16.24	28.64	109.88	87.89	28.54	5539
Pusa1927-8-86	105.5	107.02	16.78	26.52	112.92	89.64*	28.98	6760*
Pusa1927-13-55	106.5	105.29	15.55	29.07	108.59	89.60*	28.67	5698
Pusa1927-45-72	105.0	107.56	15.80	27.84	109.92	91.52*	29.23	6371
Pusa1927-62-2	107.0*	113.03*	13.95	27.82	109.38	89.28*	27.49	5811
Pusa1927-56-20	105.5	111.75*	15.50	29.00	110.23	88.19	28.37	6691
Pusa1927-75-56	107.5*	109.41	19.22*	28.66	117.97*	88.93	28.64	6079
Pusa1927-62-42	106.5	111.14	15.95	27.50	114.51	85.60	28.96	6058
PB1121	105.4	107.33	15.03	28.57	108.44	85.29	28.88	5997
CD (0.05)	1.36	3.96	2.94	2.08	6.31	3.38	1.96	591

^*^Significance at 5%.

DFF: Days to 50% flowering, PH: Plant height, TN: Tiller number, PL: Panicle length, FGP : Filled grains/panicle, SF: Spikelet fertility, TGW: Thousand grain weight, YLD: Plot yield.

**Table 3:** Grain and cooking quality, reaction to BB isolates and RPG recovery of the BC_2_F_5_ NILs carrying *Xa38* in the genetic background of PB1121.

Genotype	KLBC (mm)	KBBC (mm)	L/B	KLAC (mm)	KBAC (mm)	KER	Aroma	% RPG recovery	BB lesion length (cm)	
									Ludhiana	Kaul
Pusa1927-19-21	8.54	1.60^*^	5.35	17.60^*^	2.34	2.06^*^	2	94.7	1.5	2.0
Pusa1927-14-77	8.63	1.67	5.18	17.95^*^	2.32	2.08^*^	2	96.21	1.0	1.4
Pusa1927-17-16	8.54	1.63	5.24	17.61^*^	2.32	2.06^*^	2	96.21	0.2	1.4
Pusa1927-8-86	8.67	1.63	5.34	18.16	2.19	2.10	2	94.7	0.1	1.0
Pusa1927-13-55	8.42	1.67	5.05	18.22	2.38	2.17	2	96.21	0.2	0.9
Pusa1927-45-72	8.73	1.70	5.14	19.00	2.28	2.18	2	93.18	0.7	1.0
Pusa1927-62-2	8.73	1.67	5.23	18.22	2.53	2.09^*^	2	96.92	1.0	0.5
Pusa1927-56-20	8.62	1.70	5.08	18.32	2.58	2.13	2	95.45	1.2	2.0
Pusa1927-75-56	8.74	1.67	5.25	19.16^*^	2.32	2.20	2	96.92	1.0	1.8
Pusa1927-62-42	8.53	1.67	5.13	18.78	2.78^*^	2.20	2	96.21	1.0	1.8
PB1121	8.65	1.67	5.21	18.62	2.29	2.20	2		20.1	22.4
CD (0.05)	0.30	0.04	0.18	0.48	0.43	0.10				

**Table 4:** Reaction of NILs carrying BB resistance genes *Xa38* or *xa13 + Xa21* in the genetic background of PB1121 against six different races of Xoo.

Xoo strains	Pusa1927-75-56	Pusa1927-56-20	Pusa1927-45-72	Pusa1718-1	Pusa1718-2	Pusa1718-3	Pusa Basmati 1121
	PB1121* *+* Xa38*			PB1121 + *xa13 + Xa21*			
Race 1	1.00 ± 0.31	1.03 ± 0.29	1.73 ± 0.64	1.63 ± 0.09	1.30 ± 0.06	1.37 ± 0.19	16.07 ± 0.59
Race 2	0.70 ± 0.15	1.37 ± 0.32	1.27 ± 0.19	2.10 ± 0.21	1.87 ± 0.20	1.40 ± 0.10	16.30 ± 1.94
Race 4	15.00 ± 1.38	12.33 ± 1.09	16.50 ± 0.76	15.60 ± 0.57	13.57 ± 0.79	14.87 ± 1.32	18.83 ± 3.66
Race 5	2.07 ± 0.72	0.77 ± 0.15	3.83 ± 0.64	9.37 ± 0.26	9.40 ± 0.25	10.30 ± 0.55	16.23 ± 1.32
Race 6	2.43 ± 1.30	0.30 ± 0.10	0.40 ± 0.10	6.60 ± 0.10	6.03 ± 0.09	6.87 ± 0.20	21.40 ± 0.53

## References

[b1] Singh V. P., Singh A. K., Atwal S. S., Joseph M. & Mohapatra T. Pusa 1121: a rice line with exceptionally high cooked kernel elongation and basmati quality. International Rice Research Notes, 27(1), 25–26 (2002).

[b2] APEDA, India export of agro food products: Product group report/country wise—Basmati rice. Accessed online from: http://agriexchange.apeda.gov.in on 25 March 2016.

[b3] Ellur R. K. . Improvement of Basmati rice varieties for resistance to blast and bacterial blight diseases using marker assisted backcross breeding. Plant Sci. 242, 330–341 (2016). 26566849 10.1016/j.plantsci.2015.08.020

[b4] Jones J. D. & Dangl J. L. The plant immune system. Nature 444, 323–329 (2006). 17108957 10.1038/nature05286

[b5] Nurnberger T., Brunner F., Kemmerling B. & Piater L. Innate immunity in plants and animals: striking similarities and obvious differences. Immunol. Rev. 198, 249–66 (2004). 15199967 10.1111/j.0105-2896.2004.0119.x

[b6] Alfano J. R. & Collmer A. Type III secretion system effector proteins: double agents in bacterial disease and plant defense. Annu. Rev. Phytopathol. 42, 385–414 (2004). 15283671 10.1146/annurev.phyto.42.040103.110731

[b7] White F. F. & Yang B. Host and pathogen factors controlling the rice *Xanthomonas oryzae* interaction. Plant Physiol. 150, 1677–86 (2009). 19458115 10.1104/pp.109.139360 PMC2719118

[b8] Mudgett M. B. New insights to the function of phytopathogenic bacterial TypeIII effectors in plants. Annu. Rev. Plant Biol. 56, 509–31 (2005). 15862106 10.1146/annurev.arplant.56.032604.144218

[b9] Park C. & Ronald P. C. Cleavage and nuclear localization of the rice XA21 immune receptor. Nat. Commun. 3, 920, doi: 10.1038/ncomms1932 (2012). 22735448 PMC3621455

[b10] Song C. & Yang B. Mutagenesis of 18 Type III effectors reveals virulence function of XopZpxo99 in *Xanthomonas oryzae* pv. *oryzae*. Mol. Plant Microbe Interact. 23, 893–902 (2010). 20521952 10.1094/MPMI-23-7-0893

[b11] Yang B. & White F. F. Diverse members of the AvrBs3/PthA family of type III effectors are major virulence determinants in bacterial blight disease of rice. Mol. Plant Microbe Interact. 17, 1192–1200 (2004). 15553245 10.1094/MPMI.2004.17.11.1192

[b12] Yang B., Sugio A. & White F. F. Avoidance of host recognition by alterations in the repetitive and C-terminal regions of AvrXa7, a type III effector of *Xanthomonas oryzae pv. oryzae*. Mol. Plant Microbe Interact. 18, 142–149 (2005). 15720083 10.1094/MPMI-18-0142

[b13] Yang B., Sugio A. & White F. F. (2006) Os8N3 is a host disease-susceptibility gene for bacterial blight of rice. PNAS. 103(27), 10503–8 (2006). 16798873 10.1073/pnas.0604088103 PMC1502487

[b14] White F. F., Potnis N., Jones J. B. & Koebnik R. The type III effectors of *Xanthomonas*. Mol. Plant Pathol. 10(6), 749–66 (2009). 19849782 10.1111/j.1364-3703.2009.00590.x PMC6640274

[b15] Mondal K. K. . Pathotyping and genetic screening of type III effectors in Indian strains of *Xanthomonas oryzae* pv. *oryzae* causing bacterial leaf blight of rice. Physiol. and Mol. Plant Pathol. 86, 98–106 (2014).

[b16] Adhikari T. B., Basnyat R. C. & Mew T. W. Virulence of *Xanthomonas oryzae* pv. *oryzae* on rice lines containing single resistance genes and gene combinations. Plant Dis. 83, 46–50 (1999). 10.1094/PDIS.1999.83.1.46 30845439

[b17] Shanti M. L. . Understanding the Bacterial blight pathogen- combining pathotyping and molecular marker studies. Int. J. Plant Pathol. 1 **(2)**, 58–68 (2010).

[b18] Mishra D. . Pathotype and genetic diversity amongst Indian isolates of *Xanthomonas oryzae* pv. *oryzae*. PLOS One. 8(11), e81996. doi: 10.1371/journal.pone.0081996 (2013). 24312391 PMC3843720

[b19] Kim S. M. . Identification and fine–mapping of a new resistance gene, *Xa40*, conferring resistance to bacterial blight races in rice (*Oryza sativa* L.). Theor. Appl. Genet. 128, 1933–1943 (2015). 26081948 10.1007/s00122-015-2557-2

[b20] Hutin M., Sabot F., Ghesquière A., Koebnik R. & Szurek B. A knowledge-based molecular screen uncovers a broad-spectrum OsSWEET14 resistance allele to bacterial blight from wild rice. The Plant J. 84 **(4)**, 694–703 (2015). 26426417 10.1111/tpj.13042

[b21] Joseph M. . Combining bacterial blight resistance and Basmati quality characteristics by phenotypic and molecular marker-assisted selection in rice. Mol. Breed. 13, 377–387 (2004).

[b22] Gopalakrishnan S. . Integrating marker assisted background analysis with foreground selection for identification of superior bacterial blight resistant recombinants in Basmati rice. Plant Breed. 127, 131–139 (2008).

[b23] Basavaraj S. H. . Marker-assisted improvement of bacterial blight resistance in parental lines of PusaRH10, a superfine grain aromatic rice hybrid. Mol. Breed. 26, 293–305 (2010).

[b24] Cheema K. K. . A novel bacterial blight resistant gene from *Oryza nivara* mapped to 38 kb region on chromosome 4L and transferred to *Oryza sativa* L. Genet. Res. Cambridge. 90, 397–307 (2008). 19061530 10.1017/S0016672308009786

[b25] Bhasin H. . New PCR-based sequence-tagged site marker for bacterial blight resistance gene *Xa38* of rice. Mol. Breed. 30, 607–611 (2012).

[b26] Murray M. G. & Thompson W. F. Rapid isolation of high molecular weight plant DNA. Nucleic Acids Res. 8 **(19)**, 4321–4325 (1980). 7433111 10.1093/nar/8.19.4321 PMC324241

[b27] Kauffman H. E., Reddy A., Hsieh S. P. Y. & Merca S. D. An improved technique for evaluating resistance of varieties to *Xanthomonas oryzae* pv. *oryzae*. Plant Dis. Rep. 57, 537–541 (1973).

[b28] Khanna A. . Development and evaluation of near–isogenic lines for major blast resistance gene(s) in Basmati rice. Theor. Appl. Genet. 128 **(7)**, 1243–1259 (2015). 25869921 10.1007/s00122-015-2502-4

[b29] Dhaliwal H. S. & Kular J. S. Package of practices for the crops of Punjab (ed. Kular J. S.) 2–3 (Punjab Agricultural University, Ludhiana, 2014). Available at http://www.kvkfaridkot.com/pp_kharif_2014.pdf (Accessed: 10^th^ November, 2015).

[b30] Singh A. . SNP haplotypes of the BADH1 gene and their association with aroma in rice (Oryza sativa L.) Mol. Breed. 26, 325–338 (2010).

[b31] Bradbury L. M. T., Fitzgerald T. L., Henry R. J., Jin Q. S. & Waters D. L. E. The gene for fragrance in rice. Plant Biotechnol. J. 3, 363–370 (2005). 17129318 10.1111/j.1467-7652.2005.00131.x

[b32] Singh V. K. . Marker-assisted simultaneous but stepwise backcross breeding for pyramiding blast resistance genes *Pi2* and *Pi54* into an elite Basmati rice restorer line PRR78. Plant Breed. 132 **(5)**, 486–495 (2013).

[b33] Singh V. K. . Incorporation of blast resistance into “PRR78”, an elite Basmati rice restorer line, through marker assisted backcross breeding. Field Crops Res. 128, 8–16 (2012).

[b34] Vikal Y. . Identification of new sources of bacterial blight (*Xanthomonas oryzae* pv. *oryzae*) resistance in wild *Oryza* species and *O*. *glaberrima*. Plant Genet. Resour. C. 5, 108–112 (2007).

